# Induction of Combination Therapy for the Management of Hepatitis C: An Observational Study

**DOI:** 10.7759/cureus.10259

**Published:** 2020-09-05

**Authors:** Sultan Z Khan, Muhammad Umar Talha, Bushra Iftikhar, Amina Noor, Talha Laique, Aamna Latif, Jahanzeb Malik

**Affiliations:** 1 Gastroenterology, Abbottabad International Medical College, Abbottabad, PAK; 2 Internal Medicine, District Headquarter Hospital, Gujranwala, PAK; 3 Biochemistry, Azra Nadeed Medical College, Lahore, PAK; 4 Internal Medicine, Abbottabad International Medical College, Abbottabad, PAK; 5 Pharmacology, Lahore Medical and Dental College, Lahore, PAK; 6 Hematology, Ahmed Medical Complex, Rawalpindi, PAK; 7 Cardiology, Rawalpindi Institute of Cardiology, Rawalpindi, PAK

**Keywords:** hepatitis c, ribavirin, rapid virological response, interferon

## Abstract

Background and objective

Hepatitis C infection is prevalent in Pakistan. The purpose of this study was to observe the therapeutic effects of conventional interferon in combination with ribavirin among treatment-naive hepatitis C patients.

Methods

This descriptive cross-sectional study of hepatitis C combination therapy was conducted at our institute after approval. All the patients received treatment with conventional interferon (5-MU three times weekly) and ribavirin (1000mg/day) for four weeks. A follow-up for the rapid virological response (RVR) was done in the fourth week of treatment.

Results

The mean age of the patients was 37.43. There was a gradual decrease in RVR with increasing age after four weeks of treatment.

Conclusion

The combination therapy showed good RVR in the fourth week among all hepatitis C patients.

## Introduction

The hepatitis C virus (HCV) penetrates all body fluids, including the cerebrospinal fluid [1]. It is transmitted in many ways that include the use of unsterilized syringes, blood donation, the use of unscreened blood products, and transplanted organs. It is usually asymptomatic initially but presents as fatigue, body aches, and fever [2]. Approximately 13% of the patients develop liver cirrhosis or hepatic carcinoma if untreated [3].

Even though the exact mechanism of action of antiviral drug ribavirin remains exploratory, the current gold standard treatment option for HCV patients is its use in combination with interferon-based therapies [4-5].

Therapeutic failure happens in most HCV patients for reasons such as an inappropriate dosing regimen, non-compliance due to adverse effects, and lack of awareness of disease consequences. Some patients, having resistant HCV genotypes fail to respond to antiviral medications [6].

Two types of pegylated interferons have been developed until now. They both differ in their pharmacokinetic, pharmacodynamic, and chemical properties. Although pegylated interferon demonstrated better results and efficacy when compared with conventional interferons, it is not cost-effective in developing countries [7].

The only solution to prevent disease progression is viral eradication. In Pakistan, conventional interferon is used because pegylated interferon is unaffordable to most patients. Combined therapy increases the viral response rate as compared to interferon given as monotherapy [8]. We conducted this study to see the effect of conventional interferon and ribavirin on rapid virological response (RVR) in patients with treatment-naive HCV patients.

## Materials and methods

This was an observational study. It was carried out from January 2020 to July 2020 after approval by the ethical committee. The calculated sample size was 132 following the methodology adopted in a previous study with a modification. The treatment given was conventional interferon (5-MU three times weekly) and ribavirin (1000 mg/day) for four weeks to all enrolled patients. RVR with follow-up at the fourth week of treatment was done. Both genders with an age range between 20 and 45 years were included. Patients with pre-existing liver diseases, malignancy, pregnancy females, those taking immune-suppressant drugs, previous HCV-treated patients, and those who refused to give informed consent were excluded from the project. All participants of the study gave written informed consent at enrollment time.

Age (in years) was presented as mean±S.D. Parameters like sex and RVR presented as frequency and percentages. RVR was stratified among age and gender to see the effect modifications. Data analysis was done using the Statistical Package for the Social Sciences (SPSS) version 26 (IBM Corp, Armonk, NY).

## Results

In this study, 132 patients were enrolled. Their age, gender distribution, and RVR are shown in Table 1.

**Table 1:** Baseline characteristics and RVR RVR: rapid virological response; undetectable serum hepatitis C virus (HCV) ribonucleic acid (RNA) level at week four of treatment (RVR)

Baseline characteristics and RVR	
Age (years) mean±SD	37.43 + 4.73
Gender n(%)	
Females	46 (34.8%)
Males	86 (65.1%)
RVR n(%)	
Total	115 (87.1%)
Males	79 (91.8%)
Females	36 (78.3%)

Out of 132 patients, 115 (87.1%) achieved RVR with the combination therapy. The response was more in males as compared to females (91.8% vs. 78.3%, p=0.001).

## Discussion

The major objective of the treatment of treatment-naive chronic HCV patients is to prevent progression to cirrhosis and thereby decrease morbidity and mortality. The combination of interferon and ribavirin is effective and achieves a sustained virological response in 50% of patients [9]. Conventional interferon therapy in combination with ribavirin is more effective than interferon or ribavirin alone in the treatment of HCV infected patients. However, RVR was similar when compared with pegylated interferon plus ribavirin therapy in several studies [10-11].

Interferon-based therapy can assist in identifying patients by predicting a rapid response to therapy. Thus, it can provide information to interrupt treatment if patients are either non-responders or showing a plateau in virologic response. In this way, we can save patients from experiencing adverse events and an undue cost of treatment. In the current study, 87% of enrolled patients showed RVR to conventional interferon plus ribavirin by Week 4 of treatment. In these patients, the absence of HCV RNA in serum very likely indicated the eradication of the chronic HCV infection and the subsequent interruption of disease progression, with a low risk of further relapse or development of cirrhosis. In patients with chronic hepatitis, previous studies have shown similar results in sustained virologic response but in our region, this was the first study to evaluate combination therapy among HCV patients for RVR [12-13].

The absence of detectable HCV RNA is consistent with the view that HCV infection may be cleared with antiviral therapy even in chronic hepatitis patients. This is similar to the results reported in one study, which shows partial clearance of 44% of HCV RNA [14]. The presence of a low level of HCV RNA in the liver, not detected on PCR, cannot be ruled out because the technique used in this study was not sufficiently sensitive. In our study, however, at the end of treatment and during follow-up, no relapse was seen in patients achieving RVR in the majority of the patients (87.1%).

There were a few limitations to this study: (i) it was conducted in a single center and the sample size was small; and (ii) the time to follow-up was only four weeks, which could be prolonged for more accurate results.

## Conclusions

In conclusion, this is the first study in low socioeconomic settings at our institute to show that persistent suppression of HCV replication can be obtained early after treatment and patients may be cured of the disease by combination therapy. The absence of detectable HCV RNA at four weeks in patients with chronic treatment-naive HCV seems to be a reliable indicator of long-term virological response. Further trials should be encouraged at our centers to accurately describe the effectiveness of combination therapy, as it may reduce the burden of disease and cost on non-affording patients.

## References

[1] Garozzo A, Falzone L, Rapisarda V (2017). The risk of HCV infection among health-care workers and its association with extrahepatic manifestations (review). Mol Med Rep.

[2] Marcellin F, Di Beo V, Aumaitre H (2020). Patient-reported symptoms during direct-acting antiviral treatment: a real-life study in HIV-HCV coinfected patients (ANRS CO13 HEPAVIH). J Hepatol.

[3] Nilsson E, Anderson H, Sargenti K, Lindgren S, Prytz H (2016). Incidence, clinical presentation and mortality of liver cirrhosis in Southern Sweden: a 10-year population-based study. Aliment Pharmacol Ther.

[4] Reichard O, Norkrans G, Frydén A, Braconier JH, Sönnerborg A, Weiland O (1998). Randomised, double-blind, placebo-controlled trial of interferon alpha-2b with and without ribavirin for chronic hepatitis C. Lancet.

[5] Samuel D, Bizollon T, Feray C (2003). Interferon-alpha 2b plus ribavirin in patients with chronic hepatitis C after liver transplantation: a randomized study. Gastroenterology.

[6] Pawlotsky JM, Germanidis G, Neumann AU, Pellerin M, Frainais PO, Dhumeaux D (1998). Interferon resistance of hepatitis C virus genotype 1b: relationship to nonstructural 5A gene quasispecies mutations. J Virol.

[7] Siebert U, Sroczynski G, Rossol S (2003). Cost effectiveness of peginterferon alpha-2b plus ribavirin versus interferon alpha-2b plus ribavirin for initial treatment of chronic hepatitis C. Gut.

[8] Schalm SW, Weiland O, Hansen BE (1999). Interferon-ribavirin for chronic hepatitis C with and without cirrhosis: analysis of individual patient data of six controlled trials. Eurohep Study Group for Viral Hepatitis. Gastroenterology.

[9] Lai MY (2006). Combined interferon and ribavirin therapy for chronic hepatitis C in Taiwan. Intervirology.

[10] Yu JW, Wang GQ, Sun LJ, Li XG, Li SC (2007). Predictive value of rapid virological response and early virological response on sustained virological response in HCV patients treated with pegylated interferon alpha-2a and ribavirin. J Gastroenterol Hepatol.

[11] Martinot-Peignoux M, Maylin S, Moucari R (2009). Virological response at 4 weeks to predict outcome of hepatitis C treatment with pegylated interferon and ribavirin. Antivir Ther.

[12] Waheed Y (2015). Effect of interferon plus ribavirin therapy on hepatitis C virus genotype 3 patients from Pakistan: treatment response, side effects and future prospective. Asian Pac J Trop Med.

[13] Chen JY, Lin CY, Wang CM (2011). IL28B genetic variations are associated with high sustained virological response (SVR) of interferon-α plus ribavirin therapy in Taiwanese chronic HCV infection. Genes Immun.

[14] Lam NP, Neumann AU, Gretch DR, Wiley TE, Perelson AS, Layden TJ (1997). Dose-dependent acute clearance of hepatitis C genotype 1 virus with interferon alfa. Hepatology.

